# Study on the correlation between plasma concentration of voriconazole and clinical efficacy and safety in elderly patients

**DOI:** 10.3389/fphar.2025.1726902

**Published:** 2026-01-05

**Authors:** Yueran Li, Huifang Xu, Huifang Wang, Wen Zhang, Yujia Song, Sheng Wang

**Affiliations:** Department of Pharmacy, The First Affiliated Hospital of Wannan Medical College (Yijishan Hospital of Wannan Medical College), Wuhu, Anhui, China

**Keywords:** clinical efficacy, elderly patients, safety, therapeutic drug monitoring, voriconazole

## Abstract

**Objective:**

To investigate the relationship between voriconazole plasma concentration, clinical efficacy, and adverse reactions in elderly patients.

**Methods:**

A retrospective analysis was conducted on the clinical data of 37 elderly patients with invasive fungal infections who received voriconazole therapy and underwent therapeutic drug monitoring. The associations between voriconazole plasma concentration, treatment efficacy, and safety were evaluated.

**Results:**

After voriconazole treatment, the levels of liver function markers (ALT, AST, GGT, TBIL) significantly increased, while levels of albumin, WBC, neutrophils (NEUT), CRP, and PCT significantly decreased in elderly patients. Voriconazole plasma concentration was negatively correlated with albumin, calcium, and WBC, and positively correlated with ALT, AST, CRP, and PCT (P < 0.05). Multivariate analysis identified glucocorticoid use as an independent factor influencing voriconazole concentration (P < 0.05). No significant association was found between voriconazole concentration and dosage or clinical efficacy (P > 0.05), but a significant correlation was observed with the incidence of hepatotoxicity (P = 0.042), with an optimal predictive threshold of 4.5 μg/mL (AUC = 0.858).

**Conclusion:**

Voriconazole plasma concentrations in elderly patients vary widely, and concentrations exceeding 4.5 μg/mL do not enhance efficacy but are associated with an increased risk of hepatotoxicity, warranting close monitoring in clinical practice.

## Introduction

Invasive fungal infections (IFIs) are a type of infectious disease caused by fungi invading the body and proliferating within tissues, organs, or the bloodstream. Individuals with compromised immune function, particularly those with hematologic malignancies, organ transplant recipients, and elderly critically ill patients, exhibit significantly higher incidence and mortality rates (Maertens et al., 2021). Voriconazole (VCZ), a broad-spectrum triazole antifungal agent, is recommended by domestic and international guidelines as a first-line treatment for invasive fungal infections, fluconazole-resistant *Candida* species, and rare fungal infections due to its potent antifungal activity against *Aspergillus* and *Candida* pathogens (Patterson et al., 2016; Chen et al., 2018; Liang et al., 2022).

However, VCZ exhibits nonlinear pharmacokinetics in humans, with plasma concentrations influenced by multiple factors including age, hepatic and renal function, inflammatory status, genetic polymorphisms, and drug interactions, leading to significant interindividual variability that affects therapeutic efficacy and safety (Li et al., 2020). As the dosage increases, plasma concentrations rise significantly. Studies have shown that VCZ plasma concentrations are closely associated with treatment outcomes and adverse effects (Jin et al., 2016; Chen et al., 2018; Shen et al., 2022; Takesue et al., 2022). Common adverse effects include hepatotoxicity, visual disturbances, and neurotoxicity, with hepatotoxicity rates of 12.5%–46.7% in Asian populations, and 27.8% in Chinese populations (Zhou et al., 2020; Wang et al., 2022). Therefore, close monitoring of liver function is essential during treatment. Due to its narrow therapeutic window and interindividual variability, clinical application of VCZ faces challenges, and therapeutic drug monitoring (TDM) is considered a critical measure for achieving individualized dosing. Guidelines recommend incorporating TDM into routine management to maintain plasma concentrations within the 0.5–5 μg/mL range, balancing efficacy and safety (Chen et al., 2018).

Although the clinical value of TDM is widely recognized, existing studies predominantly focus on non-elderly adults, with limited evidence on its application in elderly populations (John et al., 2019; Abdul-Aziz et al., 2020). Elderly patients, characterized by physiological decline, comorbidities, and polypharmacy, are more prone to plasma concentration fluctuations, increasing the risk of treatment failure or adverse effects (Cheng et al., 2020). Currently, there is no consensus on the optimal therapeutic window for VCZ or standardized TDM protocols for elderly patients. To explore the rational use of VCZ in the elderly, this study retrospectively analyzed clinical records of elderly patients with invasive fungal infections treated with VCZ, assessing the impact of gender, age, concomitant medications, and laboratory parameters on plasma concentrations, as well as risk factors for adverse effects. This analysis aims to provide a reference for individualized treatment strategies in this population.

## Data and methods

### Data source

A single-centre retrospective analysis of data from adults (≥60 years old) with ≥1 voriconazole trough measured at the First Affiliated Hospital of Wannan Medical College (Yijishan Hospital of Wannan Medical College), Wuhu, Anhui Province, China from 2023 to 2024 was performed. A retrospective data collection was conducted on elderly patients treated with voriconazole and undergoing therapeutic drug monitoring at the First Affiliated Hospital of Wannan Medical College from January 2023 to December 2024. Inclusion criteria were: age ≥60 years, continuous voriconazole treatment for ≥3 days, and at least one steady-state serum trough concentration measurement. Patients were excluded if they lacked complete laboratory evaluation data. This study was approved by the Ethics Committee of the First Affiliated Hospital of Wannan Medical College (Yijishan Hospital of Wannan Medical College) (protocol number: 2025-ky-129), and strictly followed the research design. All methods were carried out in accordance with the guidelines and regulations of the First Affiliated Hospital of Wannan Medical College (Yijishan Hospital of Wannan Medical College). Due to the retrospective nature of the study, Scientific Research and New Technology of Wannan Medical College Yijishan Hospital IRB waived the need of obtaining informed consent.

### Data collection

In this study, relevant clinical data were collected through the hospital information system. The data included patients’ baseline characteristics, such as sex, age, body weight, and clinical diagnosis; laboratory parameters, including including serum albumin (ALB), liver and renal function indicators, C-reactive protein (CRP), white blood cell count (WBC), neutrophil count (NEUT), platelet count (PCT), and serum electrolyte levels; voriconazole dosing information, including dosage, administration route, treatment duration, and steady-state serum concentrations; and data on concomitant medications, such as glucocorticoids, proton pump inhibitors (PPIs), statins, diuretics, and antimicrobial agents.

### Therapeutic drug monitoring of voriconazole blood concentrations

Steady-state voriconazole trough concentrations were monitored as follows: for patients who received a loading dose, blood was sampled 30 min prior to the administration on day 3 of therapy. For patients who did not receive a loading dose, blood was sampled before the administration between days 5 and 7 of treatment. Venous blood was collected in EDTA-anticoagulated tubes (2 mL), followed by centrifugation at 3,000 r/min for 5 min. The supernatant (200 μL) was used for subsequent analysis. The therapeutic drug monitoring in this study specifically measured the trough concentration of the parent drug voriconazole.

Plasma voriconazole concentrations were measured using an established two-dimensional liquid chromatography system (GI-3000D). The system configuration included: two GI-3000-P04 quaternary pumps and one GI-3000-P01 single pump; a GI-3000-A01 large-volume autosampler; a GI-3000-F01 column oven; and a GI-3000-D01 UV detector. Chromatographic separation was performed on a Waters C18 analytical column (100 mm × 4.6 mm, 5 µm), coupled with an extraction column (4.0 mm I.D. × 20 mm). The mobile phases consisted of: Pump 1-(A) methanol and (B) water; Pump 2-(A) acetonitrile, (B) methanol, (C) buffer salt, and (D) water; Pump 3-(A) water. The detection wavelength was set at 255 nm, the column temperature was maintained at 30 °C, and the injection volume was 50 µL. Voriconazole concentrations were quantified using the external standard method with the GI-3000 chromatography data processing software.

The analytical method was fully validated according to the Guidelines for Bioanalytical Method Validation. The lower limit of quantification was 0.5 μg/mL, and the upper limit of quantification was 20.0 μg/mL. The method demonstrated an accuracy of 96.58%. The intra-day precision, expressed as the relative standard deviation (RSD), was 0.13% for retention time and 0.5% for peak area. The inter-day precision RSD was 0.54% for retention time and 0.72% for peak area. This validated method ensures the reliability and reproducibility of all voriconazole plasma concentration data reported in this study.

### Adverse reaction assessment

Common adverse reactions associated with voriconazole include hepatotoxicity, electrolyte disturbances, and neurotoxicity. Compared to other antifungal agents, voriconazole has a higher propensity to induce liver toxicity and visual-related symptoms (Xing et al., 2017; Liu et al., 2024). To systematically evaluate the incidence and severity of hepatic injury, patient case data were meticulously reviewed and analyzed. Specifically, liver function parameters during treatment, namely, alanine aminotransferase (ALT), aspartate aminotransferase (AST), alkaline phosphatase (ALP), gamma-glutamyl transpeptidase (GGT), and total bilirubin (TBIL) were recorded in detail. The degree of hepatotoxicity caused by voriconazole was graded according to the Common Terminology Criteria for Adverse Events (CTCAE) version 5.0, with Grades 3 and 4 classified as severe hepatic impairment (Table 1) (Freites-Martinez et al., 2021; Taghvaye-Masoumi et al., 2021; Cheng et al., 2024b). Neuropsychiatric adverse events, including delirium, visual or auditory hallucinations, agitation, excitation, and irritability, were actively screened for through retrospective review of all clinical notes and nursing records.

**TABLE 1:** Hepatic dysfunction grades as described by NCI (17).

Feature	Grade 1	Grade 2	Grade 3	Grade 4
ALT	>1.0–2.5	>2.5–5.0	>5.0–20	>20
AST	>1.0–2.5	>2.5–5.0	>5.0–20	>20
ALP	>1.0–2.5	>2.5–5.0	>5.0–20	>20
GGT	>1.0–2.5	>2.5–5.0	>5.0–20	>20
TBIL	>1.0–1.5	>1.5–3.0	>3.0–10	>10

The values expressed the multiples of the upper limit of the normal range (ULN). ALT: alanine transaminase; AST: aspartate aminotransferase; ALP: alkaline phosphatase; GGT: Gamma-glutamyl transferase; TBIL: total bilirubin; NCI: national cancer institute.

Moreover, previous studies have indicated that voriconazole treatment may lead to electrolyte disturbances, such as hypokalemia and hyponatremia (Alawfi et al., 2022). Therefore, electrolyte imbalance was also considered an adverse effect indicator in this study. The diagnostic criteria for electrolyte disturbances were serum potassium levels <3.5 mmol/L and serum sodium levels <135 mmol/L. The severity was categorized into three levels: mild, with serum potassium between 3.0 and 3.5 mmol/L and serum sodium between 130 and 135 mmol/L; moderate, with potassium between 2.5 and 3.0 mmol/L and sodium between 125 and 130 mmol/L; and severe, with potassium <2.5 mmol/L and sodium <125 mmol/L.

### Evaluation of efficacy

The assessment of voriconazole’s therapeutic effectiveness should be conducted through a multidimensional approach that integrates clinical, radiological, and mycological indicators. Clinically, the evaluation primarily focuses on the improvement of infection-related symptoms, including the resolution of fever, changes in affected sites (such as the lungs), and the dynamic variation of inflammatory markers (e.g., CRP). Regular imaging examinations, such as chest X-rays and pulmonary computed tomography (CT) scans, are essential for monitoring the progression or resolution of pulmonary lesions. From a mycological perspective, ongoing measurement of serum galactomannan antigen levels serves to reflect the fungal burden. If, during treatment, at least two of these three assessment criteria show no improvement or worsen, it should be considered a lack of significant response to voriconazole therapy. The use of these comprehensive evaluation methods facilitates a thorough and scientific judgment of voriconazole’s antifungal efficacy, thereby providing robust clinical evidence to guide treatment decisions.

### Statistical analysis

All statistical analyses were performed using SPSS version 26.0. Continuous variables with a normal distribution were expressed as mean ± standard deviation (x̄±s), and comparisons between groups were conducted using the independent t-test. Categorical variables were described as frequencies and percentages, with intergroup comparisons performed using the chi-square (χ^2^) test. For continuous variables with a non-normal distribution, data were presented as medians with interquartile ranges [M (P25, P75)], and group differences were assessed using the Mann-Whitney U test. Further comparisons of categorical variables were carried out using Fisher’s exact test. In univariate analyses, potential predictive variables were identified using Pearson’s chi-square test. Subsequently, both one-way ANOVA and multivariate logistic regression models were employed to explore key factors influencing voriconazole serum concentrations and adverse events. Receiver operating characteristic (ROC) curve analysis was used to evaluate the sensitivity, specificity, and optimal cutoff values of potential risk factors as predictors. All statistical analyses were conducted using R software and the Python environment, with a P-value of <0.05 considered statistically significant.

### Ethical considerations

This study was done under the ethical approval of The First Affiliated Hospital of Wannan Medical College (Yijishan Hospital of Wannan Medical College) Human Research Ethics Committee (HREC) for using clinical database, reference number:Approval No.: 2025-ky-129.

## Results

### Patient characteristics and voriconazole plasma concentrations

Data from 98 patients who received voriconazole therapy and underwent therapeutic drug monitoring were collected from the Hospital Information System (HIS). Based on the inclusion and exclusion criteria, a final dataset of blood concentration measurements was obtained for 37 elderly patients, comprising 25 males and 12 females, with a mean age of 74.54 ± 6.66 years. The frequency of therapeutic drug monitoring (TDM) varied according to clinical requirements. The median number of voriconazole plasma trough concentration measurements per patient was 1, with a range of 1–4. The majority of patients (78.4%) underwent a single measurement, typically obtained at the initial steady-state assessment. Repeat measurements (2–4 per patient) were performed in a subset of patients (21.6%), primarily in cases requiring dose adjustment, suspected toxicity, or reevaluation of treatment response. Relevant demographic and clinical characteristics, including a detailed summary of TDM frequency, are detailed in Table 2. All 37 patients were diagnosed with or suspected of having invasive fungal infections. According to the EORTC/MSGERC criteria, 18.92% (7/37) had proven, 45.95% (17/37) had probable, and 35.14% (13/37) had presumed invasive fungal infections. The most common pathogens identified were *Candida spp.* (32.43%) and *Aspergillus spp.* (24.32%). Infections involved the respiratory tract (89.19%), central nervous system, and urinary tract. A detailed breakdown of pathogens and infection sites is provided in Supplementary Table S1.

**TABLE 2:** Demographic and clinical characteristics of patients (n = 37).

Characteristics	Values
Sex	
Male, n (%)	25 (67.57)
Female, n (%)	12 (32.43)
Age (years)	74.54 ± 6.66
Weight (kg)	62.04 ± 6.12
Comorbidities [n (%)]	
Diabetes mellitus	9 (24.32)
Chronic obstructive pulmonary disease	8 (21.62)
Chronic kidney disease	9 (24.32)
Solid tumor	8 (21.62)
Malnutrition	5 (13.51)
Bronchiectasis	5 (13.51)
Autoimmune disease	4 (10.81)
Route of administration	
Intravenous, n (%)	25 (67.56)
Oral, n (%)	6 (16.22)
Switch from IV to oral therapy, n (%)	6 (16.22)
Combined use of antibiotics, n (%)	
Tetracyclines	3 (8.1)
Beta-lactam antibiotics	29 (78.38)
Quinolones	16 (43.24)
Glycopeptide antibiotics	9 (24.32)
Carbapenems	10 (27.03)
Combined use of glucocorticoid, n (%)	21 (56.76)
Combined use of PPIs, n (%)	25 (67.57)
Combined use of diuretic, n (%)	22 (59.46)
Combined use of statins, n (%)	6 (16.22)
Combined use of hepatic enzyme inducers, n (%)	6 (16.22)
Combined use of hepatoprotective agents, n (%)	13 (35.14)
VCZ plasma concentration (μg/mL)	4.21 (0.65,12.59)
VCZ dose (mg/kg/day)	6.05 ± 1.42
Number of VCZ trough level measurements per patient	1 (1,4)

### The correlation between voriconazole plasma concentration and dosage

**FIGURE 1:**
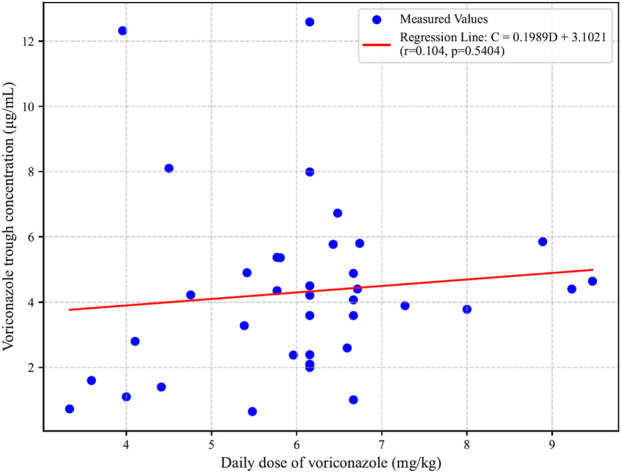
Correlation between voriconazole trough concentrations and daily dose.

Data collected from the hospital information system (HIS) indicated that the median voriconazole trough concentration was 4.21 μg/mL (range: 0.65–12.59 μg/mL). Among the patients, 27% (n = 10) had trough concentrations exceeding the upper limit of the therapeutic window (5 μg/mL), while no measurements fell below the lower limit (0.5 μg/mL). The mean daily dose of voriconazole was 6.05 ± 1.42 mg/kg. Correlation analysis revealed no statistically significant relationship between voriconazole trough concentrations and its dosage (p = 0.54, r = 0.1) (Figure 1).

### Dose adjustment based on voriconazole therapeutic drug monitoring

In clinical practice, voriconazole plasma concentrations occasionally exceed the therapeutic window, prompting dose adjustments based on therapeutic drug monitoring (TDM) results. This retrospective analysis identified 10 patients with trough concentrations above the upper limit of the therapeutic window (5 μg/mL). Among these, six patients (60%) underwent dose reduction, consistent with the goal of minimizing toxicity risk. For the remaining four patients (40%), clinicians opted to maintain the current dose, a decision likely influenced by individual patient factors such as disease severity, clinical response, and drug tolerability.

### Changes in laboratory parameters before and after treatment

This study utilized data from the hospital HIS system to collect relevant laboratory parameters before and after voriconazole administration in the enrolled patients. Paired *t*-tests were performed to compare parameters related to liver function, renal function, electrolytes, complete blood count, and inflammatory markers. The results indicated that serum albumin levels, a marker of liver function, significantly decreased from 31.8 ± 4.77 g/L before treatment to 29.76 ± 4.03 g/L after treatment (P = 0.002). Additionally, the mean levels of ALT, AST, GGT, and TBIL were significantly elevated during treatment compared to baseline (P_ALT_ = 0.028, P_AST_ < 0.001, P_GGT_ = 0.004, P_TBIL_ = 0.027). For complete blood count parameters, both white blood cell (WBC) count and neutrophil (NEUT) count showed decreasing trends. WBC count declined from 11.06 ± 5.41 × 10^9^/L pre-treatment to 9.28 ± 5.87 × 10^9^/L post-treatment (P = 0.001), while NEUT count decreased from 9.68 ± 5.32 × 10^9^/L to 7.73 ± 5.96 × 10^9^/L (P < 0.001). Regarding inflammatory markers, both C-reactive protein (CRP) and procalcitonin (PCT) levels were significantly lower after voriconazole therapy compared to baseline, suggesting a potential impact of voriconazole on inflammatory responses. Voriconazole treatment also significantly affected renal function, with notable changes observed in renal function indices (P < 0.001). In contrast, electrolyte levels did not show significant differences before and after treatment. Detailed information on the specific changes in these laboratory parameters is provided in Table 3.

**TABLE 3:** Effect of voriconazole on relevant laboratory indicators.

Parameters	Pre-treatment	Post-treatment	P
ALB (g/L)	31.8 ± 4.77	29.76 ± 4.03	0.002
ALT (U/L)	21.45 ± 12.61	61.35 ± 75.42	0.028
AST (U/L)	41.64 ± 70.64	80.67 ± 131.54	0.000
GGT (U/L)	53.9 ± 73.94	115.9 ± 139.76	0.004
TBIL (μmol/L)	15.25 ± 12.67	16.37 ± 26.57	0.027
Cr (μmol/L)	116.96 ± 110.62	103.13 ± 67.89	0.000
K (mmol/L)	4.11 ± 0.55	4.01 ± 0.6	0.147
Na (mmol/L)	139.79 ± 5.93	143.37 ± 8.91	0.412
Ca (mmol/L)	8.45 ± 24.41	1.99 ± 0.23	0.918
WBC (10^9^/L)	11.06 ± 5.41	9.28 ± 5.87	0.001
NEUT (10^9^/L)	9.68 ± 5.32	7.73 ± 5.96	0.000
PCT (g/L)	167.73 ± 76.22	149.6 ± 101.91	0.000
CRP (mg/L)	100.59 ± 87.02	46.01 ± 60.62	0.039

Alkaline phosphatase was excluded from the liver function analysis due to incomplete data.

### Correlation analysis between laboratory parameters and voriconazole plasma concentrations before and after treatment

Laboratory parameters before and after voriconazole administration were extracted from the hospital HIS system for the enrolled patients. Pearson correlation analysis was performed to assess the relationships between voriconazole plasma concentrations and various clinical laboratory parameters, including liver and renal function indices, electrolytes, complete blood count, and inflammatory markers. The results showed that none of the baseline (pre-treatment) laboratory parameters exhibited a significant correlation with voriconazole plasma concentrations (P > 0.05). However, after treatment, serum albumin, serum calcium, and WBC count were significantly negatively correlated with voriconazole plasma concentrations (P < 0.05). In contrast, ALT, AST, CRP, and PCT levels were significantly positively correlated with voriconazole concentrations (P < 0.05). Detailed correlation coefficients and statistical values are presented in Table 4.

**TABLE 4:** Correlation analysis between laboratory parameters and voriconazole plasma concentrations before and after treatment.

Parameters	Pre-treatment		Post-treatment	
	P	R	P	R
ALB	0.541	0.109	0.039	−0.355
ALT	0.923	−0.021	0.005	0.549
AST	0.344	−0.189	0.014	0.467
GGT	0.129	0.319	0.191	0.277
TBIL	0.618	0.11	0.059	0.399
Cr	0.105	−0.313	0.354	−0.182
K	0.390	−0.193	0.300	−0.231
Na	0.664	−0.076	0.913	−0.019
Ca	0.411	−0.162	0.034	−0.401
WBC	0.611	0.103	0.045	−0.388
NEUT	0.908	0.022	0.080	−0.325
PCT	0.120	0.29	0.041	0.375
CRP	0.010	0.45	0.004	0.498

### Univariate and multivariate analyses of factors influencing voriconazole plasma concentrations

Univariate regression analysis identified glucocorticoid use as the only factor significantly associated with voriconazole plasma concentrations (P = 0.012, R^2^ = 0.167). Serum creatinine (Cr) levels approached statistical significance (P = 0.085; Table 5), suggesting a potential relationship between renal function and voriconazole clearance, warranting further investigation in multivariate analysis. In addition, voriconazole treatment-related variables identified in the univariate analysis were included in a multivariate logistic regression model. The results demonstrated that glucocorticoid use remained a significant independent predictor of elevated voriconazole concentrations (P < 0.05; Table 6). Serum creatinine levels and treatment duration showed borderline significance, indicating that these factors may also be clinically relevant and should not be overlooked in therapeutic monitoring.

**TABLE 5:** Univariate analysis of factors influencing voriconazole plasma concentrations.

Variable	Coefficient	P	R^2^
Demographic characteristics			
Age	−0.044	0.301	0.031
Male	0.039	0.949	0.000
Weight	−0.017	0.721	0.003
Cr	−0.007	0.085	0.082
Voriconazole medication information			
Voriconazole dose	0.318	0.127	0.065
Treatment duration	0.043	0.116	0.069
Complete blood count			
CRP	0.002	0.687	0.005
PCT	−0.001	0.792	0.002
WBC	0.011	0.766	0.003
NEUT	0.019	0.360	0.024
Combination therapy			
Hepatic enzyme inducer	0.260	0.736	0.003
Glucocorticoid	1.375	0.012	0.167
PPIs	0.246	0.685	0.005
Statins	−0.518	0.499	0.013
Diuretic	0.524	0.362	0.024
Carbapenems	0.419	0.510	0.013
Beta-lactam antibiotics	0.381	0.580	0.009
Tetracyclines	−0.336	0.746	0.003
Glycopeptide antibiotics	−0.044	0.947	0.000
Quinolones	−0.783	0.166	0.054

**TABLE 6:** Multivariate logistic regression analysis of factors associated with voriconazole plasma concentrations.

Variable	OR (95% CI)	Coefficient	P
Glucocorticoid	5.120 (2.009,13.050)	1.633	0.001
Cr	0.993 (0.986,1.000)	−0.007	0.059
Treatment duration	1.046 (1.000,1.094)	0.045	0.050
Voriconazole dose	1.274 (0.880,1.845)	0.242	0.192

### Impact of voriconazole plasma concentrations on efficacy

The relationship between voriconazole (VCZ) plasma concentrations and treatment efficacy was evaluated in 37 elderly patients over a median treatment duration of 12 days (range: 3–57 days). Patients were categorized into effective and ineffective groups based on clinical outcomes, and according to the voriconazole trough concentration at the time of assessment, patients were further stratified into two groups: a normal concentration group (defined as trough levels within 0.5–5 μg/mL) and a high concentration group (defined as trough levels exceeding 5 μg/mL). Data analysis showed that 44.44% (12 patients) in the normal concentration group achieved effective outcomes, compared to 60.00% (6 patients) in the high concentration group (Table 7). Due to the limited sample size, Fisher’s exact test was used, yielding a P value of 0.476. This result suggests that, within the observed treatment period, higher VCZ plasma concentrations do not significantly improve antifungal efficacy.

**TABLE 7:** Analysis of the association between voriconazole plasma concentrations and treatment efficacy.

Curative effect	Normal-concentration cases	High-concentration cases	P
Ineffective	15	4	0.476
Effective	12	6	
Total	27	10	

### Voriconazole-induced hepatotoxicity and its associated risk factors

A total of 25 patients (67.57%) experienced hepatotoxicity during voriconazole therapy. The incidences of grade ≥1 adverse events for alanine aminotransferase (ALT), aspartate aminotransferase (AST), γ-glutamyl transpeptidase (GGT), and total bilirubin (TBIL) were 40.54%, 59.46%, 67.57%, and 13.51%, respectively. No grade 4 liver injury was observed. The grading of voriconazole-induced hepatotoxicity is shown in Table 8.

**TABLE 8:** Grading and number of cases of voriconazole-induced hepatotoxicity.

Parameters	Classification of hepatic injury			Total
	Grade 1	Grade 2	Grade 3	
ALT	10	4	1	15
AST	18	1	3	22
GGT	13	5	7	25
TBIL	1	1	3	5

**FIGURE 2:**
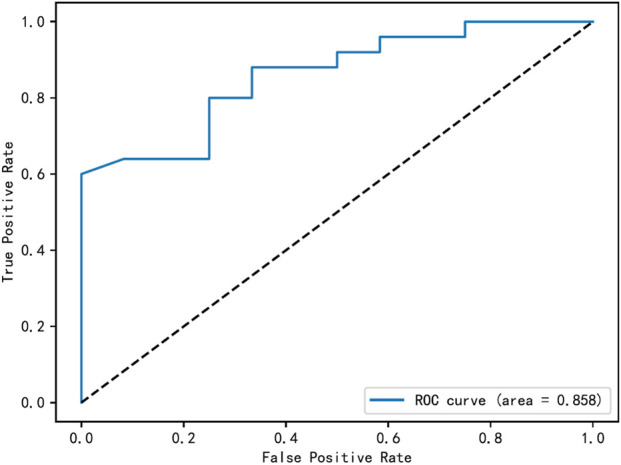
ROC curves of voriconazole trough concentrations to predict liver injury in elderly patients.

Univariate analysis indicated that daily dose of voriconazole, duration of therapy, and plasma concentration were significantly associated with hepatotoxicity (all P < 0.05; Table 9). These three variables were then included as independent variables in a multivariate logistic regression model. The corresponding odds ratios (ORs) and 95% confidence intervals (CIs) were: daily dose, OR = 2.06 (95% CI: 0.74–5.71, P = 0.164); duration of therapy, OR = 1.10 (95% CI: 0.95–1.27, P = 0.195); and plasma concentration, OR = 2.37 (95% CI: 1.03–5.45, P = 0.042). These results suggest that elevated voriconazole plasma concentration is an independent risk factor for hepatotoxicity. The area under the receiver operating characteristic (ROC) curve (AUC) for plasma concentration in predicting hepatotoxicity was 0.858, with an optimal cutoff value of 4.5 μg/mL (sensitivity: 60.0%, specificity: 100.0%), as shown in Figure 2.

**TABLE 9:** Univariate analysis of risk factors for voriconazole-related adverse events.

Variable	Hepatic injury			Electrolyte imbalance		
	Yes (n = 25)	No (n = 12)	P	Yes (n = 13)	No (n = 24)	P
Demographic characteristics						
Age [year, Mean ± SD]	74.00 ± 5.36	75.67 ± 8.98	0.484	74.62 ± 5.09	74.50 ± 7.48	0.961
Male [n (%)]	16 (64)	9 (75)	0.711	10 (76.92)	15 (62.5)	0.476
Weight [kg, Mean ± SD]	61.42 ± 7.21	63.33 ± 2.46	0.380	61.73 ± 6.72	62.21 ± 5.90	0.656
Voriconazole medication information						
Voriconazole dose [mg/kg/d, Mean ± SD]	6.47 ± 1.34	5.42 ± 1.14	0.034	6.16 ± 1.38	6.11 ± 1.37	0.974
Treatment duration [d, Mean ± SD]	17.24 ± 11.30	10.08 ± 6.43	0.039	16.00 ± 9.17	14.33 ± 11.23	0.473
Voriconazole plasma concentration [μg/mL, Mean ± SD]	4.75 ± 1.46	2.73 ± 1.29	0.000	3.98 ± 1.62	4.16 ± 1.76	0.762
Complete blood count						
CRP [mg/L, Mean ± SD]	72.68 ± 70.03	76.25 ± 93.41	0.935	76.22 ± 70.90	72.55 ± 81.65	0.578
PCT [g/L, Mean ± SD]	139.60 ± 100.30	149.25 ± 100.17	0.786	115.23 ± 85.19	157.63 ± 104.32	0.245
WBC [×10^9^, Mean ± SD]	10.02 ± 8.15	9.32 ± 6.15	0.793	8.75 ± 9.99	10.36 ± 5.89	0.119
NEUT [×10^9^, Mean ± SD]	11.80 ± 16.56	7.30 ± 6.65	0.373	13.27 ± 22.74	8.75 ± 6.11	0.578
Combination therapy [n (%)]						
Hepatic enzyme inducer	3 (12)	3 (25)	0.367	2 (15.38)	4 (16.67)	1.000
Glucocorticoid	16 (64)	5 (41.67)	0.291	7 (53.85)	14 (58.33)	1.000
PPIs	18 (72)	7 (58.33)	0.468	11 (84.62)	14 (58.33)	0.149
Statins	2 (8)	4 (33.33)	0.073	2 (15.38)	4 (16.67)	1.000
Diuretic	16 (64)	6 (50)	0.488	5 (38.46)	17 (70.83)	0.083
Carbapenems	8 (32)	2 (16.67)	0.445	5 (38.46)	5 (20.83)	0.275
Beta-lactam antibiotics	19 (76)	10 (83.33)	1.000	9 (69.23)	20 (83.33)	0.413
Tetracyclines	1 (4)	2 (16.67)	0.241	2 (15.38)	1 (4.17)	0.278
Glycopeptide antibiotics	8 (32)	1 (8.33)	0.220	4 (30.77)	5 (20.83)	0.691
Quinolones	9 (36)	7 (58.33)	0.291	5 (38.46)	11 (45.83)	0.739

### Electrolyte imbalances observed during voriconazole therapy

A total of 13 patients developed electrolyte disturbances while on voriconazole therapy, primarily hypokalemia in 8 cases (21.62%) and hyponatremia in 7 cases (18.92%). Two patients experienced both hypokalemia and hyponatremia simultaneously. Regarding severity grading, hypokalemia was mostly mild (6 cases, 75.00%) and moderate in 2 cases (25.00%), with no severe hypokalemia observed. Similarly, hyponatremia was predominantly mild (6 cases, 85.71%) and moderate in 1 case (14.29%). Univariate analysis showed no significant associations between the risk of voriconazole-induced electrolyte disturbances and demographic characteristics, voriconazole treatment parameters, or concomitant medications (P > 0.05; Table 9).

### Neurotoxicity during voriconazole therapy

Through a retrospective review of medical records, one patient (2.7%) was found to have developed neurotoxicity during voriconazole treatment, presenting with hallucinations and delirium. At the time of the event, the monitored plasma concentration of voriconazole was 7.99 μg/mL. The symptoms resolved after discontinuation of the drug and administration of supportive care.

## Discussion

Current research on voriconazole therapeutic drug monitoring and individualized dosing strategies primarily focuses on invasive fungal infections and solid organ transplant recipients, with most patients being under 60 years of age (Marks et al., 2017; Maertens et al., 2021; Boutin et al., 2024). Studies specifically investigating voriconazole TDM in elderly patients (≥60 years) remain limited. This retrospective study analyzed the plasma concentrations, efficacy, and adverse reactions of voriconazole in 37 elderly patients, exploring the correlation between drug levels and clinical outcomes. Hamada et al. reported a median trough concentration of 3.9 μg/mL in patients receiving adequate voriconazole dosing (including loading doses), with 30% exhibiting high trough concentrations (≥5 μg/mL) (Hamada et al., 2020). In our study, the median trough concentration following adequate dosing was 4.21 μg/mL, and 27% of patients had trough levels >5 μg/mL, which is lower than the 4.7 μg/mL median reported by Park et al., where 38% of patients exhibited high trough concentrations (≥5.5 μg/mL) (Park et al., 2012). These discrepancies may be attributed to differences in sample size and disease severity among the included patients. Notably, elderly patients often exhibit decreased hepatic and renal function as well as reduced metabolic enzyme activity, leading to decreased voriconazole clearance.

Previous studies have demonstrated that voriconazole plasma concentrations increase with age; even with conventional dosing, elderly patients have 60%–90% higher trough concentrations compared to younger individuals (Allegra et al., 2020). Patients over 60 years old show significantly higher plasma levels than younger patients (Cheng et al., 2020), and population pharmacokinetic models confirm reduced clearance in the elderly (Wang et al., 2024). Additionally, age-related CYP2C19 genetic polymorphisms, such as poor metabolizer status, may further contribute to increased variability in drug concentrations (Zhang et al., 2021). Differences in the reference target concentration ranges for voriconazole may also account for some inconsistencies across studies. Previous studies have demonstrated that voriconazole pharmacokinetics in adults exhibit nonlinear behavior and considerable interindividual variability, influenced by multiple factors (Veringa et al., 2023). Our study found that similar large variability in voriconazole plasma concentrations exists among elderly patients, with levels ranging from 0.65 to 12.59 μg/mL. Albumin, serum calcium, and white blood cell counts showed significant negative correlations with voriconazole plasma concentrations, while ALT, AST, CRP, and PCT exhibited significant positive correlations. Additionally, drug-drug interactions play a crucial role in voriconazole metabolism. Voriconazole is primarily metabolized by cytochrome P450 enzymes and acts as both a substrate and inhibitor of CYP2C19, CYP2C9, and CYP3A4, making it susceptible to numerous drug interactions. Elderly patients often have multiple comorbidities and complex medication regimens, increasing the likelihood of such interactions.

This study examined the impact of various concomitant medications, including hepatic enzyme inducers (amiodarone), glucocorticoids, proton pump inhibitors, statins, diuretics, carbapenem antibiotics, β-lactam antibiotics, tetracyclines, glycopeptides, and fluoroquinolones on voriconazole plasma concentrations. Both univariate and multivariate analyses identified glucocorticoid use as a significant factor affecting voriconazole levels. Glucocorticoids are potent inducers of CYP2C9, CYP2C19, and CYP3A4 enzymes, potentially accelerating voriconazole metabolism by inducing CYP2C19 and CYP3A4 activity, thereby reducing trough concentrations (Jia et al., 2021). However, in our study, patients receiving concomitant glucocorticoids actually exhibited elevated voriconazole plasma concentrations, suggesting that metabolic pathways in elderly patients may be more complex and influenced by liver and kidney function, overall disease status, and polypharmacy. Furthermore, our findings indicate that creatinine levels and treatment duration may influence voriconazole clearance, potentially leading to drug accumulation. Given the decline in physiological function and presence of multiple comorbidities in elderly patients, factors affecting voriconazole therapy are more intricate. Therefore, therapeutic drug monitoring and individualized dosing regimens are especially important to optimize voriconazole use in this population.

There is a recognized correlation between voriconazole plasma concentrations and both clinical efficacy and toxicity, with the general consensus that voriconazole trough concentrations should be maintained within a relatively narrow therapeutic window (Luong et al., 2016). Hepatotoxicity is the most common adverse reaction associated with voriconazole, and its pathogenesis is mainly related to oxidative stress and lipid metabolism disturbances (Wu et al., 2019). Previous studies have reported that abnormal elevation of serum liver enzymes is common during voriconazole therapy, with incidence rates ranging from 13.4% to 69% (Tverdek et al., 2016). In our study, the incidence of voriconazole-related liver injury was as high as 67.57%, suggesting that elderly patients may have a higher risk of hepatotoxicity compared to the general adult population. Voriconazole-related liver injury in this cohort predominantly manifested as abnormal liver function tests, mainly elevations in ALT, AST, and GGT, with fewer cases showing increased total bilirubin (TBIL). Severity assessment indicated that liver injury was mostly mild, and most patients had a favorable prognosis, consistent with previous domestic reports (Shen et al., 2022).

Previous studies have identified factors influencing voriconazole-related hepatotoxicity, including hypoalbuminemia, voriconazole plasma concentrations, and CYP2C19 genetic polymorphisms (Xiao et al., 2023). In this study, univariate analysis indicated that treatment duration, daily dose, and plasma concentration differences were statistically significant; however, multivariate logistic regression analysis demonstrated that only plasma concentration was an independent risk factor. ROC curve analysis identified a voriconazole trough concentration of 4.5 μg/ml as a critical threshold associated with increased liver injury risk. Similarly, a retrospective study by Hirata A et al. found that voriconazole trough concentration was an independent risk factor for hepatotoxicity, with a cutoff value of 4.2 μg/mL (Hirata et al., 2019). The critical trough concentration identified in our study is consistent with these findings. Additionally, as plasma concentration and area under the curve (AUC) increase, the risk of hepatotoxicity correspondingly rises (Hanai et al., 2021). Regarding efficacy, our data did not show a significant correlation between higher voriconazole plasma concentrations and improved antifungal outcomes (Table 7). Therefore, while a minimum concentration is necessary for efficacy as established in prior literature, exceeding the upper threshold appears to increase toxicity risk without conferring additional therapeutic benefit in our elderly cohort. Taken together, our findings suggest that in elderly patients, controlling the voriconazole trough concentration, particularly maintaining it below approximately 4.5 μg/mL, is crucial for minimizing hepatotoxicity risk. When integrated with the established efficacy threshold from guideline recommendations, this safety upper limit may help inform a more personalized and safer therapeutic range for this vulnerable population.

Voriconazole can also cause electrolyte disturbances, such as hypokalemia and hyponatremia. However, the risk factors for developing hypokalemia and hyponatremia remain unclear. Higher doses and elevated voriconazole plasma concentrations are generally considered contributing factors for hypokalemia (Benitez and Carver, 2019). Cheng L et al. reported incidences of hypokalemia and hyponatremia at 18.0% and 7.9%, respectively, mostly of mild severity (Cheng et al., 2024a). In our study, the incidences of voriconazole-induced hypokalemia and hyponatremia were 21.62% and 18.92%, respectively, also predominantly mild cases, which is consistent with the above findings. Cheng L et al. found through multivariate analysis that no independent risk factors were identified for hyponatremia, whereas sex, the ratio of voriconazole N-oxide trough concentration to voriconazole trough concentration, and concomitant antibiotic use were independent risk factors for hypokalemia. In contrast, our univariate analysis did not reveal any significant indicators associated with voriconazole-induced electrolyte disturbances. This may be due to the complex mechanisms underlying these adverse reactions, involving multiple drug interactions and underlying comorbidities.

Voriconazole treatment may induce central nervous system (CNS) toxicity, with clinical manifestations including hallucinations, delirium, and visual disturbances. Its occurrence is closely associated with elevated plasma concentrations, and a higher trough level is considered a major risk factor for neurotoxicity. Yang L et al. found that the incidence of neurotoxicity was significantly correlated with the voriconazole trough concentration, with a risk threshold of approximately 4.85 μg/mL (Yang et al., 2022). When the plasma concentration exceeded 4.85 μg/mL, the incidence of neurotoxicity increased significantly to 32.9%. The study by Shen K et al. also indicated that the median voriconazole trough concentration in the neurotoxicity group was 5 μg/mL, which was significantly higher than that in the group without adverse events, and neurotoxicity was one of the common reasons for dose adjustment (Shen et al., 2022). In the present study, one patient (2.7%) was found to have developed neurotoxicity, presenting with hallucinations and delirium. The plasma concentration at the time of the event was 7.99 μg/mL, which is above the risk threshold reported in the aforementioned studies. The symptoms resolved after drug discontinuation and supportive care. The incidence of neurotoxicity in our study was lower than that reported in the literature, which may be attributed to the smaller sample size, differences in population characteristics, and limitations of the retrospective assessment method. Current research generally agrees that elevated plasma concentrations are a key driver of neurotoxicity, although the precise mechanism may involve voriconazole crossing the blood-brain barrier and affecting neurotransmitter systems or ion channel function (e.g., TRPM1/TRPM3) (Yang et al., 2022). In clinical practice, routine therapeutic drug monitoring for patients receiving voriconazole and maintaining trough concentrations within a safe range are crucial measures for preventing neurotoxicity.

In summary, this study highlights the importance of therapeutic drug monitoring of voriconazole in elderly patients during treatment. A rational TDM strategy can not only reduce unnecessary toxicity risks but also guide individualized dosing regimens. Based on both domestic and international evidence, maintaining voriconazole plasma concentrations within the range of 4–4.5 μg/mL appears to be appropriate for elderly patients. Additionally, enhanced monitoring is recommended when co-administering glucocorticoids or other concomitant medications to ensure treatment safety. This study has several limitations. First, it is a single-center retrospective analysis with a relatively small sample size. Second, due to the retrospective design, we could not confirm whether voriconazole trough concentrations had been maintained within the therapeutic range for a standardized period prior to efficacy assessment, which may affect the precision of the exposure-response analysis. Third, an observation from our therapeutic drug monitoring (TDM) practice warrants consideration as a potential source of variability in exposure-outcome analyses. Despite a predefined action threshold (>5 μg/mL), clinicians maintained the current dose in 40% of cases with supratherapeutic concentrations. This reflects the complex, real-world integration of TDM data with other patient-specific factors-such as infection severity, clinical stability, and absence of early toxicity-in the risk-benefit calculus for critically ill patients. While this represents authentic clinical decision-making, it introduces heterogeneity in exposure management that may influence the observed relationships between drug concentration and clinical outcomes. Fourth, regarding the observed electrolyte disturbances, our study design cannot establish a causative role for voriconazole, as multiple confounders (e.g., concomitant medications, underlying illness) could contribute. Fifth, although voriconazole plasma concentrations and clinical data were collected, the impact of pharmacogenetics (e.g., CYP2C19 polymorphisms) was not extensively investigated. Sixth, our TDM focused solely on the parent drug; we did not measure concentrations of the major metabolite, voriconazole N-oxide, which has been implicated in some toxicity profiles. Future research should involve larger, multicenter prospective studies that integrate genetic testing, metabolite profiling, and population pharmacokinetic modeling. Such studies would better define individualized dosing strategies, clarify the role of voriconazole in electrolyte dysregulation, and elucidate the complex relationships between metabolism, exposure, and toxicity. In conclusion, TDM remains crucial for optimizing the efficacy and safety of voriconazole therapy in elderly patients, yet its interpretation and application must be contextualized within holistic clinical assessment.

## Data Availability

The original contributions presented in the study are included in the article/Supplementary Material, further inquiries can be directed to the corresponding author.
